# Institutional challenges in responding to Austria’s Dying Decree Law: An evaluation from the perspectives of nursing and medical directors

**DOI:** 10.1177/26323524261436925

**Published:** 2026-04-26

**Authors:** Tamina-Laetitia Vielgrader, Jana Marica Hluch, Klara Doppler, Elisabeth Lucia Zeilinger, Ricarda Mewes, Sabine Parrag, Thomas Wochele-Thoma, Maria Kletecka-Pulker, Stefan Dinges

**Affiliations:** 1 Institute for Ethics and Law in Medicine, University of Vienna, Austria; 2 Vienna Doctoral School in Cognition, Behaviour and Neuroscience, University of Vienna, Austria; 3 Department of Clinical and Health Psychology, Faculty of Psychology, University of Vienna, Austria; 4 Department of Clinical Research SBG, Academy for Ageing Research, Haus der Barmherzigkeit, Vienna, Austria; 5 Outpatient Unit for Research, Teaching and Practice, Faculty of Psychology, University of Vienna, Austria; 6 Ludwig Boltzmann Institute Digital Health and Patient Safety, Ludwig Boltzmann Gesellschaft, Vienna, Austria

**Keywords:** assisted suicide, euthanasia, end-of-life care, healthcare policy, institutions

## Abstract

**Background:**

On 1 January 2022, assisted suicide became legal in Austria with the enactment of the Dying Decree Law (Sterbeverfügungsgesetz, StVfG). This law has posed complex practical and ethical challenges for nursing facilities and hospitals. Medical and nursing directors are tasked with guiding their staff amidst legal uncertainties, emotional burdens, and limited institutional dialogue.

**Objectives:**

This study aimed to assess directors’ self-reported knowledge of the law, their experiences managing assisted suicide requests, their perceived adequacy of support in navigating the legal framework, and the presence of institutional guidelines.

**Design:**

A cross-sectional mixed-methods study.

**Methods:**

An online questionnaire was used to collect data from 239 medical and nursing directors across all nine federal states of Austria (mean age = 49.5 years, SD = 8.5; 65.7% female, 32.2% male; 23.7% working in faith-based institutions, 61.9% in non-faith-based institutions, and 14.4% prefer not to disclose). Quantitative data were analysed using chi-square tests, *t* tests, and ANOVAs, while thematic analysis was applied to open-ended responses.

**Results:**

44.0% of directors knew the regulations of the Dying Decree Law but were insecure in regards to the application in practice, while 13.4% of directors reported no familiarity with Law at all. Legal familiarity correlated with gender (*p* = 0.020, *V* = 0.183) and institutional religious affiliation (*p* = 0.019, *V* = 0.197). Satisfaction with institutional guidelines was linked to gender (*p* = 0.004, *r* = 0.15), religious affiliation (*p* = 0.003, *r* = −0.15), and institution type (*p* = 0.011, η² = 0.126). While 36.2% reported no institutional guidelines on assisted suicide, 13.8% were unaware of their existence.

**Conclusion:**

The findings reveal knowledge gaps, institutional disparities, and limited guidance on the Dying Decree Law. Directors report insufficient preparedness, highlighting the need for clearer institutional guidelines and enhanced legal, ethical, and psychological support.

**Trial registration:**

This study was registered in the Open Science Framework (OSF) at https://osf.io/bgpsa.

## Plain language summary

How prepared are healthcare directors in Austria to deal with assisted suicide requests?

In January 2022, assisted suicide became legal in Austria under a new law called the Dying Decree Law. This change created new responsibilities and challenges for healthcare institutions such as hospitals and nursing homes. Medical and nursing directors play a key role in helping staff respond to requests for assisted suicide, but little was known about how well prepared they feel to do this.

This study explored how familiar medical and nursing directors in Austria are with the new law, how confident they feel applying it in practice, and whether clear institutional guidelines on assisted suicide are available. An online survey was completed by 239 medical and nursing directors from all nine Austrian federal states. The survey included multiple-choice questions and open-ended responses to capture both numerical data and personal experiences. The findings show that many directors feel uncertain about the law. While some were familiar with the legal rules, nearly half felt insecure about how to apply them in real situations, and some had no knowledge of the law at all. Knowledge and satisfaction with institutional guidance differed depending on gender, whether the institution was faith-based, and the type of healthcare setting. More than one third of directors reported that their institution had no guidelines on assisted suicide, and others were unsure whether such guidelines existed.

Overall, the study highlights important gaps in knowledge, guidance and support. Many directors feel unprepared to manage assisted suicide requests and the emotional and ethical challenges involved. The results suggest a clear need for better legal information, ethical guidance, psychological support, and clear institutional policies. Improving national coordination and institutional structures could help ensure consistent, ethical, and high-quality end-of-life care in Austria.

## Introduction

End-of-life autonomy has been a topic of public debate in several countries for decades. There is continued, broad-based discussion on whether ending one’s own life should be allowed in specific circumstances and within a legally secured environment.^1^ These ethical debates have been fuelled by social conversations and legal disputes concerning respect for autonomy, compassion and relief from suffering, safeguards and regulations, suicide prevention and patient rights.^1,2^

In December 2020, the Austrian Constitutional Court ruled that a prohibition of every form of assistance in suicide is unconstitutional, and obliged the legislator to take action. This ruling led to the *Sterbeverfügungsgesetz* (Dying Decree Law) being introduced on 1 January 2022.^3^ This short time frame may have limited the opportunity to develop a broad, organic social and national consensus on assisted suicide in Austria, a phenomenon also observed in several Latin American countries.^4^

The Dying Decree Law states that the person wishing to die (PWTD) must meet the following conditions: (a) must be at least 18 years old; (b) must have an incurable terminal or severe chronic illness that greatly limits their quality of life; (c) must be an Austrian citizen or a permanent resident of Austria; and (d) must be capable of making decisions.

**Figure 1.**
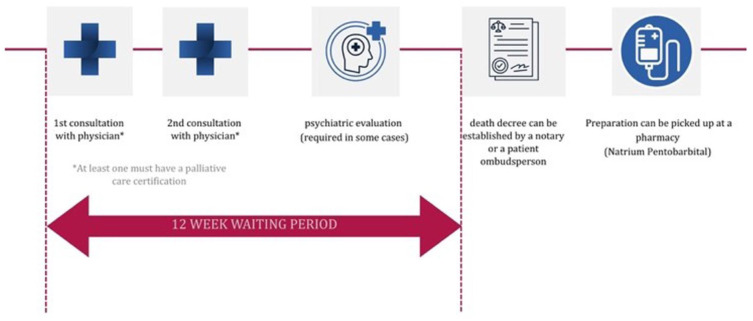
Process of assisted suicide in Austria. This figure was designed by the authors to illustrate the steps involved in the process.

Persons meeting these conditions must go through a rigorous process (see Figure 1) including a 12-week waiting period (that may be shortened if the terminal stage has already been reached) and a medical consultation by at least two physicians, one of whom must have palliative care certification. After the waiting period, a dying decree can be issued by either a notary or a patient ombudsperson. This decree records the free and self-determined death of the PWTD and documents the process up to the establishment.^5^

The consultations should determine whether the person making the request meets the required conditions, and inform the patient of alternatives (palliative care, end-of-life care, etc.).

And lastly, the law clearly states that the final step (drinking the preparation or opening the IV line) must be taken by the PWTD, as Austria only permits assistance in suicide, and not euthanasia in the form of physician-administered death. Furthermore, any involvement in assisted suicide, whether providing consultation, assisting or dispensing the lethal preparation, is entirely voluntary, and no individual can be compelled to participate.^5^

The legal stipulation that all involvement must be voluntary has had a marked consequence: assisted suicides are increasingly shifted to the private sphere. While the reasons for this shift remain insufficiently documented and cannot be attributed to clinicans’ conscientious objection alone, the current legal and institutional framework appears to displace assisted suicide from regulated healthcare settings into the private sphere, where oversight is minimal, limited structural guidance and data collection are severely impeded.^6^ It is essential to note that there is no assessment of the process, respectively, meaning that no authority verifies whether everything proceeded as the legislation had intended. Moreover, there is no *point-of-contact* or information centre that provides services or advocacy regarding assisted suicide in Austria, leaving PWTD and their relatives alone in a bureaucratic maze.

Nonetheless, many end-of-life requests are often first voiced within healthcare institutions. Literature from various countries has demonstrated that this situation places professionals in a challenging position, characterised by legal uncertainty, emotional strain, and conflicting ethical obligations.^7–11^ Nursing and medical directors are confronted with the challenge of guiding staff through wishes to die and assisted suicide requests based on limited legal clarity, while simultaneously attempting to guarantee professional responsibility and patient autonomy.

Although the legal text gives a clear outline of the necessary steps to establish a dying decree, neither political bodies nor medical professional organisations have so far provided explicit operational directives to healthcare institutions. Consequently, due to the limited legal framework governing the management of data and support for assisted suicide requests in Austria, medical and nursing directors face numerous practical challenges in their management roles.^12^ These include providing leadership and supervision, overseeing medical quality standards, fulfilling legal and ethical obligations towards patients and employees, and ensuring that medical practice aligns with institutional policy and healthcare regulations. They also play a vital role in assessing and supporting the performance and professional development of healthcare staff within their institutions. In this context an Austrian study provide the most relevant empirical reference point, showing that there is no online statement of palliative care or hospice institution that supports assisted suicide, and that there is only a minority who have provided online statements on assisted suicide, most of which were dominantly Christian and opposed Assisted suicide (AS).^13^ These findings suggest that institutional positions on assisted suicide are largely restrictive and may constitute a structural framework within which individual professionals operate. Against this backdrop, examining the perspectives of medical and nursing directors allows insight into how institutional stances are interpreted, negotiated, or enacted at the leadership level.

Our study aims to provide an overview of how nursing and medical directors perceive the legislation regulating assisted suicide, the obstacles they face when confronted with requests for assisted suicide, and the guidelines they have or have not implemented to help healthcare professionals in their respective institutions navigate requests for assisted suicide.

This study is pioneering as it is the first nationwide research on the influence of the Dying Decree Law on medical and nursing directors in general. By using sociodemographic variables as covariates, the study aims to provide insights into trends and possible patterns in bureaucratic and organisational challenges related to dying decrees.

The existing body of research on assisted suicide in Austria (since 1 January 2022) has largely been conducted by palliative care physicians, psychologists, and legal academics. The existing research focuses on the relationship between palliative care and assisted suicide, the legal interpretation of the Dying Decree Law, and the correlation between assisted suicide and suicide prevention and reveals a gap in sociocultural analyses and in the inclusion of patient, family, and broader public narratives.^5,13–16^ By identifying these dominant disciplinary perspectives, we aim to situate our research within the existing discourse and reveal potential gaps in sociocultural or experiential approaches to assisted suicide.

Furthermore, while there is some research on the moral and ethical attitudes of assisted suicide among physicians and nurses, there remains insufficient research to date on the complex role, challenges and responsibilities of hospitals, and nursing facilities in responding to such requests.^13–15,17–22^ Hereby, it is important to note that the Austrian Federal Ministry of Labour, Social Affairs, Health, Care and Consumer Protection has published a Guide for Practice regarding assisted suicide. While this guide is intended to provide orientation aid for all individuals involved in the process, it also lacks clear instructions.^23^

Based on the identified points of interest, the present study addresses the following five main research questions: (1) How do medical and nursing directors at Austrian hospitals and nursing facilities assess their own understanding of the Dying Decree Law, and how confident do they feel in its application? (2) What experiences have medical and nursing directors had with requests for assisted suicide, and how have they addressed or managed these situations in practice? (3) How frequently are internal guidelines available within institutions, and how do medical and nursing directors evaluate their quality in terms of usefulness? (4) To what extent do medical and nursing directors report information deficits and changes needed to enhance the practicability of the Dying Decree Law? (5) Do gender, institutional religious affiliation, and type of healthcare facility have an impact on medical and nursing directors’ familiarity with the law, the provision of related guidelines, and their evaluation of guideline quality?

## Materials and methods

We used a cross-sectional study design in the form of an online mixed-methods survey targeted at Austrian medical and nursing directors to examine nursing and hospital management understanding and attitudes towards the Dying Decree Law (Supplemental Material: Additional File 1).

### Study design and materials

This study employs a mixed-methods design with both exploratory and explanatory components. Findings from prior international studies^24–27^ and the expertise of the research team informed the development of the questionnaire. The research team included professionals with backgrounds in medicine, medical law and ethics, social science, public health, and psychology. The survey consisted of 24 items, most of which were designed to be answered in the format of quantitative scales. The exploratory dimension of the study allowed for the identification of novel themes and contextual insights, particularly as medical and nursing directors have not previously been the focus of research on assisted suicide requests. Simultaneously, the quantitative component enabled the identification and explanation of patterns in institutional practices, perceptions, and procedural frameworks. A summary of the questionnaire structure, including item categories and scaling, is presented in Table 1.

**Table 1.** Summary of the measurement items in the questionnaire.

Category	No. of items	Description	Scaling
Sociodemographic data	7	Federal state, religious affiliation, and type of institution in which the physician works, as well as the physician’s age, gender, length of employment at the respective institution, and the number of patients in their care	Multiple choice, single choice, and open format
Knowledge and understanding of the Dying Decree Law	1	Attitude to the law in general	Single choice
Encounters and experiences with assisted suicide	11	Experiences, contact, and situations related to the wish to die (within regard to the framework of the Dying Decree Law)	Yes/no questions, multiple choice and single choice, open format
Institutional guidelines	3	Satisfaction with the guidelines, existence of guidelines	Yes/no questions and Likert scale
Need for change and guidance	2	Information needed, practicability of the law	Yes/no question, open format, multiple choice

The structure, phrasing, and content of the items were reviewed by experts in medicine, medical law and ethics, social science, public health, psychology and palliative care to ensure clarity and relevance, and assess content and face validity before distribution. Based on their feedback, wording and structure were refined. The questionnaire was not pilot tested with the target population. The questionnaire was available for a period of 3 months, from August 2024 to mid-October 2024.

The questionnaire was specifically developed for this study by authors T.L.V. and J.M.H. and has not been previously published. An English-language version of the questionnaire is provided as “Supplemetal Material: Additional File 1.”

### Study population and procedure

Upon request, we obtained a list of all hospitals (*N* = 268) from the Federal Ministry of Labour, Social Affairs, Health, Care and Consumer Protection of the Republic of Austria^28^ and a list of all nursing facilities in Austria (*N* = 995) from Gesundheit Österreich GmbH.^29^ We reviewed and refined both lists to exclude institutions that would be incompatible with the legal requirements for assisted suicide, such as paediatric facilities or forensic psychiatric institutions.

Following this process, 1251 healthcare institutions were identified as eligible. These included institutions offering palliative care, geriatric or long-term care services, treating adult patients, and employing a medical and/or nursing director in a decision-making role. As the contact information for management staff was not consistently included in source lists, we conducted additional research or contacted each institution to obtain the email addresses of medical and nursing directors. Data collection was performed from July to September 2024.

To ensure nationwide representation, institutions from all nine Austrian federal states were contacted. Furthermore, to reflect the heterogeneity of the Austrian healthcare landscape, we included both public and private institutions in our sample. Given the public debate at the intersection between religious beliefs and assisted suicide, we also made a purposeful effort to include faith-based hospitals and care institutions. The email included a study description, an invitation to participate, and a secure link to the survey hosted on SoSciSurvey (Version 3.6.07).^30^

The first page of the questionnaire contained detailed information about the study, including its purpose, duration, and data use. Participants were informed that their responses would be fully anonymous. Additionally, participants could choose not to answer any individual question, in which case the item was recorded as missing. Consent was obtained by clicking a button on the first page. Inclusion criteria were: (i) being a nursing or medical director of an Austrian hospital or nursing facility; and (ii) having sufficient German language skills to complete the survey. Participants who did not meet these criteria were excluded. On average, survey completion took approximately 15 min.

With regards to data security, all responses were fully anonymous. The last page of the questionnaire provided participants with the option to voluntarily leave their contact information if they were willing to be considered for a potential interview within the scope of future studies.

Due to the exploratory nature of the study, no formal a priori power analysis was conducted. The study focused on descriptive analyses and identification of potential associations, and all inferential results should be interpreted as hypothesis-generating. The self-administered questionnaire assessed participants’ self-reported knowledge of the Dying Decree Law, frequency of encounters with patients regarding dying decrees and assisted suicide, availability of and satisfaction with guidelines, and perceived need for further information on assisted suicide.

### Data analysis

We used both quantitative and qualitative data. Data were exported from SoSciSurvey and analysed using JASP (Version 0.19.3).^31^ Descriptive analyses included frequencies and percentages. The significance level was set to a α = 5%. Considering the exploratory nature of the study, we did not apply alpha corrections for multiple testing. We used chi-square analysis as most of the variables (gender, institutional affiliation) were of nominal scale level. Differences in guideline quality ratings were analysed using *t* tests and ANOVA; where assumptions of normality or homogeneity of variance were violated, the Mann-Whitney *U* test was used instead.

Open-ended responses were analysed using MAXQDA. We followed the reflexive thematic analysis approach outlined by Braun and Clarke^32^ using an inductive, data-driven method. This allowed responsiveness to the complexity of the data and preserved the richness of individual perspectives. The analysis began with multiple close readings to achieve familiarity with the data. The first author (T.L.V.) maintained analytic memos during data familiarisation, coding, and theme development to document interpretive decisions, emerging assumptions, and points of uncertainty. Prior to and throughout analysis, T.L.V. and J.M.H. engaged in regular reflexive discussions in which they explicitly articulated and revisited their personal and professional positions towards assisted suicide. These ongoing exchanges functioned as a form of critical reflexive dialogue, prompting continual examination of how researchers’ perspectives might shape interpretations.

A preliminary coding tree was developed to identify overarching themes which were then refined into subcodes. We intentionally avoided predefined coding schemes to remain open to the diversity of views expressed. Throughout the process, the coding framework remained adaptable and was continuously revised based on emerging patterns. This qualitative approach reflects a pragmatic-constructivist epistemology, emphasising participants’ subjective perspectives, and the real-world complexity of institutional contexts.

Given the mixed-methods design, quantitative and qualitative data were synthesised through an integrative analytic strategy. Both data strands were analysed separately and then compared to identify areas of convergence, complementarity, or divergence. Quantitative results informed the focus of qualitative interpretation, while qualitative themes were used to explain, contextualise, and expand upon statistical findings. This integrative approach allowed triangulation of results and strengthened the interpretative validity of findings.

The questionnaire and analyses were conducted in German. Participant quotations were subsequently translated into English by a professional translator to ensure accurate representation of the intended meaning.

## Results

### Sample

One medical director and/or one nursing director in a decision-making role was invited to participate from each eligible institution. In total, 239 nursing and medical directors completed the survey, corresponding to an overall response rate of 19.1%. Of these, 157 people (65.7%) identified as female (mean age: *M* = 48.9, SD = 8.3), and 77 (32.2%) as male (mean age: *M* = 50.5, SD = 8.9), one person selected the option “prefer not to say” and four persons skipped the question regarding gender. The mean age of all nursing and medical directors was 49.5 years (SD = 8.5). On average, respondents had been employed at their current institution for 15.6 years (SD = 11.5).

### Quantitative descriptive results

#### Knowledge and understanding of the Dying Decree Law

With respect to knowledge and understanding of the Dying Decree Law, 86.6% (*n* = 207) of the 239 respondents replied that they knew the legal regulations, with 56.0% (*n* = 116) stating that they were also able to apply them in practice. The remaining 44.0% (*n* = 91) knew the regulations but felt insecure in applying them. 13.4% (*n* = 32) of the 239 participants reported having no knowledge of the regulations.

There was a significant effect between knowledge of the Dying Decree Law and gender (χ^2^(2) = 7.862, *p* = 0.020, *V* = 0.183) with male respondents reporting more often that additionally to knowing the Dying Decree Law, they were also able to apply it in practice (χ^2^(1) = 7.802, *p* = 0.005, *V* = 0.196). Self-identified gender was collected instead of sex assigned at birth to reflect participants’ own identification and potential differences in knowledge or application linked to social and cultural roles.

Moreover, a significant difference regarding familiarity was observed depending on the religious affiliation of the institution (χ^2^(2) = 7.877, *p* = 0.019, *V* = 0.197). Post hoc pairwise comparisons following the chi-square tests indicated that leadership of faith-based institutions more frequently reported higher levels of familiarity and confidence to apply the Dying Decree Law compared to leadership from non-faith-based institutions (χ^2^(1) = 7.556, *p* = 0.006, *V* = 0.242). Additionally, those who were familiar with the law but unsure how to apply its regulations in practice also differed significantly from those entirely unfamiliar with it (χ^2^(1) = 4.119, *p* = 0.042, *V* = 0.203). In contrast, the correlation between knowledge and type of institution (e.g. public/private hospital or nursing facility) fell narrowly outside the boundaries of statistical significance (see Supplemental Material: Additional File 2).

#### Encounters and experiences with assisted suicide

Medical and nursing directors were asked whether a patient had ever expressed the wish to draw up a dying decree during their stay at the respective institution: 35.6% (*n* = 83) answered “yes”; 48.9% (*n* = 114) “no”; and the remaining 15.5% (*n* = 36) were unaware of any such cases.

When asked whether they had ever encountered a patient with an established and valid dying decree in their institution, 71.8% (*n* = 163) answered “no”; 18.1% (*n* = 42) “yes”; and 10.1% (*n* = 23) were unaware.

Respondents were also asked whether patients with an established and valid dying decree had ever expressed the wish to carry out assisted suicide in their institution: 18.1% (*n* = 41) answered “yes”; 69.9% (*n* = 158) “no”; and the remaining 12.0% (*n* = 27) reported being unaware of any such cases.

As a follow-up, respondents were asked whether other individuals were involved in establishing a dying decree. Of the 46 respondents, the majority (54.4%) indicated that relatives were present throughout the process. An overview of all responses is provided in Table 2.

**Table 2.** Responses of medical and nursing directors regarding dying decrees and assisted suicides.

Question	Yes (%)	No (%)	Unaware (%)
Patient ever expressed wish to draw up a dying decree	35.6	48.9	15.5
Encountered a patient with an established and valid dying decree	18.1	71.8	10.1
Patient with valid dying decree expressed wish for assisted suicide within the institution	18.1	69.9	12.0
Presence of other individuals (e.g. relatives) during the establishment of dying decrees^a^	54.4^a^	–	–

a Based on responses from 46 individuals, this item assessed whether others were present throughout the process of assisted suicide. The item was multiple choice rather than a yes/no question.

#### Institutional guidelines

When asked about the presence of established institutional guidelines, 36.2% (*n* = 81) of the respondents answered “no” and 13.8% (*n* = 31) stated that they were unaware of the existence of in-house guidelines.

We found significant effects regarding medical and nursing home directors’ satisfaction with the guidelines depending on their gender (*U* = 1772.000, *p* = 0.004, *r* = 0.15) and the religious affiliation of the institution in which they work (*U* = 705.500, *p* = 0.003, *r* = −0.15). Female medical and nursing directors reported higher levels of satisfaction with the guidelines than male directors, and respondents working in faith-based institutions reported lower levels of satisfaction with the guidelines than those working in non-faith-based hospitals and nursing homes.

We also found significant differences in satisfaction with the guidelines depending on the type of institution in which the respondents worked (*F*(3, 45.785) = 4.178, *p* = 0.011, η² = 0.126): Managers of public hospitals reported significantly higher levels of satisfaction with the institutional guidelines than managers of private hospitals (*p* = 0.044) and nursing homes (*p* = 0.007).

The questionnaire directed respondents answering “no” to the presence of institutional guidelines (*n* = 81) to a follow-up question on whether such a guideline is currently planned. Among those who reported the absence of institutional guidelines, only 40.0% (*n* = 32) stated that guidelines are being planned, while the remaining 60.0% (*n* = 48) reported that there are currently no plans to implement a guideline on managing assisted suicide.

#### Need for change and guidance

The final two questions were designed to assess the extent to which medical and nursing home directors: (1) personally wished for more information on assisted suicide and the areas (legal, ethical and psychological) in which they saw the greatest need for assistance; and (2) whether, in their opinion, there is a general need to make Dying Decree Law more practicable, and what these changes should specifically entail.

With respect to the need for support services, 31.9% (*n* = 115) of respondents needed more legal information on the Dying Decree Law, while 30.2% (*n* =109 ) indicated a wish for more ethical information on its implementation and 17.5% (*n* = 63) were interested in further psychological guidance (it was possible to select multiple answers). 20.5% (*n* = 74) of the respondents did not require further support or information on the Dying Decree Law.

When asked whether changes were needed to make implementation of the Dying Decree Law more practicable, 66.5% (*n* = 139) of the 209 respondents who answered reported that they do not currently see a specific need for changes. Of those respondents indicating that changes are necessary (33.5%, *n* = 70), all but one (98.6%) provided concrete suggestions in a related open-ended response field. These suggestions were subsequently analysed using MAXQDA^33^ and are presented in more detail in the section “Qualitative results.”

### Qualitative results

In two open-ended questions, we asked participants (a) to share the type of support they integrated in cases of assisted suicide requests, and (b) to reflect on whether they perceived a need for changes to the current legislation.

**Figure 2.**
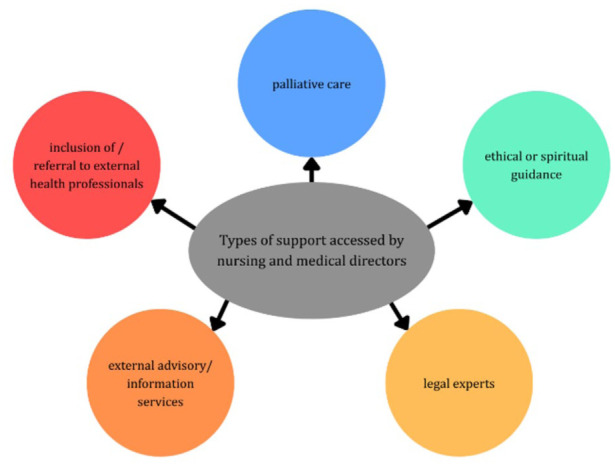
Five core themes related to types of support.

The responses revealed five core themes related to the types of support accessed by nursing and medical directors (see Figure 2). The findings reflect recurring themes from the subset of participants who provided open-ended responses and are presented as qualitative insights rather than quantitative results.

Out of the 22 participants who provided answers to the open-ended questions, several (*n* = 9 out of 22) highlighted a reliance on the expertise of palliative care specialists and mobile palliative teams. For example, participant 172 and participant 130 stated:

Palliative counselling and therapy services, multiprofessional support.

– Participant 172, Pos. 5

Taking the wish seriously and exploring its background, palliative care and counselling.

– Participant 130, Pos. 4

After palliative care, three themes were weighted equally, namely ethical/spiritual guidance, external advisory/information services, and legal experts.

Nursing and medical directors reported seeking ethics consultation services (in-house and external) for the healthcare professionals associated with the case, or spiritual or psychosocial care for patients and relatives, as exemplified by the comment made by participant 67:

Social support for the affected person and their relatives; conversations.

– Participant 67, Pos. 5

They also reported referring patients to accredited advisory services (e.g. the Austrian Medical Board, public patient advocacy institutions).

Some respondents had referred patients to general practitioners who were based out-of-house or involved external healthcare professionals (e.g. can be found on Austrian Medical Board lists for assisted suicide consultations) in cases. It should be noted that only a minority of respondents reported integrating psychiatric and/or psychological professionals in cases of requests for assisted suicide.

When asked whether changes were needed to the current framework of the Dying Decree Law, medical and nursing directors highlighted a variety of problematic areas. Firstly, most respondents questioned the practicability of the law, highlighting the lack of guidelines on how to respond to assisted suicide requests as a healthcare facility, and noted the scarcity of information on implementation in practice. Additionally, several respondents noted the challenge of responsibility: the law does not specify responsible bodies or institutions for all cases, hindering proper implementation of the law. Many respondents also reported that the legislation was written in a manner which was too complex and unintelligible to non-legal experts. This issue is specifically addressed by participants 39 and 153:

The practical procedures are not clearly regulated, and there is a lack of knowledge among all parties involved.

– Participant 39, Pos. 3

At present, many problems are left to patients and their relatives; a more professional environment would be desirable. Some formulations in the legal text are partly unrealistic (e.g. “beyond doubt” decision-making capability).

– Participant 153, Pos. 4

Across participants’ comments, we observed recurring difficulties with value-based judgements relating to the regulations of the Dying Decree Law. Participant 191 stated:

It’s not practicable and causes me moral distress.

– Participant 191, Pos. 1–4

Another key theme we identified was the challenge of bureaucratic administration. Medical and nursing directors stated that the legal and administrative procedures for assisted suicide requests were overly complex and time-consuming, placing additional strain on already limited personnel resources and burdening the (often terminally ill) patients who lacked the time or physical capacity to respond to such bureaucratic demands. This is further compounded by the fact that managing assisted suicide requests does not fall explicitly within the remit of healthcare institutions, thereby underscoring the directors’ triangular responsibility towards employees, patients, and the institution itself, without the possibility of referring the matter to a more appropriate authority.

A minority of respondents also indicated that the accumulated costs of assisted suicide can be significant for patients, with the costs differing in each federal state and often representing a financial barrier for patients wishing to pursue this option.

Finally, we identified a prevailing view among respondents, namely that the legislation overlooks certain populations. For example, nursing and medical directors noted that in its current state, the law discriminates against people with medical conditions who are often physically unable to organise and establish a dying decree or administer the lethal drug.

Comments provided by participants 215, 136, 68, and 183 clearly demonstrate the issue:

Patients with neurodegenerative diseases are severely disadvantaged.

– Participant 215, Pos. 2

Neurological patients with swallowing disorders, tetraplegia, or severe CNS developmental disorders are not taken into account.

– Participant 136, Pos. 4

People who are physically unable to carry out assisted suicide face a disadvantage.

– Participant 68, Pos. 4

Self-administration is required – discrimination against individuals with physical impairments.

– Participant 183, Pos. 3

## Discussion

This study offers critical insights into how nursing and medical directors have interpreted and navigated Austria’s Dying Decree Law since its enactment in 2022. The study findings provide significant insight into the experiences of leadership and the challenges of putting the legislation into practice within institutional settings.

We found that directors of faith-based institutions reported better knowledge of the Dying Decree Law and were more likely to be able to apply the regulations in practice than those of non-faith-based institutions. Concerning legal knowledge and understanding, our findings indicate a notable gender-based difference. Female directors are more likely to be familiar with the relevant regulations, although they more frequently report uncertainty with respect to their practical application. In contrast, male directors tend to occupy either end of the spectrum: they are more likely to report comprehensive understanding and confident application of the law or, conversely, limited or complete lack of awareness of the legislation.

Our findings show variations in legal awareness: nursing and medical directors’ engagement with regulations and jurisdiction is shaped by their institutional context (faith-based and non-faith-based) and gender. While female directors report feeling uncertain despite their knowledge of the legal text, this might reflect a cautious or relational approach to legal compliance. In contrast, the responses of male directors suggest a more dichotomous legal orientation, namely full confidence or complete detachment (Ewick and Silbey^34^).

These institutional and personal differences in legal understanding mirror the diversity of perspectives that were considered during the drafting of the Dying Decree Law. In response to the Austrian Constitutional Court’s ruling on assisted suicide in 2020, the Austrian Federal Ministry of Justice initiated the Dialogue Forum on Assisted Dying, a multi-stakeholder consultation. The forum involved participants from different backgrounds and areas of expertise (e.g. bioethics experts, government representatives, professional associations, advocacy groups), as well as from faith communities (Christian, Protestant, Muslim, Jewish and Buddhist). The forum gathered a comprehensive overview of opinions to guide legislative work following the Constitutional Court’s ruling that the criminalisation of assistance in suicide is unconstitutional.^35^

Our findings resonate with the positions of the faith communities reflected in the Dialogue Forum. Faith-based institutions – particularly the Catholic Church and associated Catholic organisations – were prominently represented and actively shaped the discourse. Their participation focused on institutional protection and ethical boundary-setting. This, in turn, may explain why directors of faith-based institutions are both more familiar with the topic and less satisfied with the existing guidelines. This reflects their active engagement in shaping policy and institutional positioning, and how they are prepared for legal and ethical navigation (although not necessarily support for assisted suicide). In line with the findings of Kitta et al.,^13^ this pattern underscores the importance of institutional context when interpreting leadership-level perceptions of assisted suicide. The largely restrictive institutional positions identified at organisational level appear to be reflected in how medical and nursing directors perceive the legislation, experience obstacles in practice, and approach the development of internal guidelines.

While the dialogue exemplifies and reflects the broader societal and ethical frameworks that guided the process of drafting the legislation, the lived realities within institutions reveal how these frameworks unfold in practice, often in ethically complex and emotionally charged situations.

As demonstrated in the findings, there is a notable imbalance between familiarity with the legislation and the ability to apply it. This theme persists across the data: the quantitative results have shown a small but very consistent fraction of respondents indicating their unawareness of key aspects of the Dying Decree Law and its implementation. Across several segments of the questionnaire, between 10% and 15% of nursing and medical directors were unaware or uncertain of the legal regulations, of cases of in-house assisted suicide requests, and of their institutional guidelines. These knowledge gaps at the leadership level may point to weaknesses in internal communication and governance, with potential implications for staff preparedness and patient outcomes.

While it is often assumed that medical and nursing directors are centrally involved in the initiation, development, and implementation of institutional guidelines, governance structures may differ substantially across healthcare institutions. In some institutions, responsibility for developing or issuing guidelines may rest with legal departments, ethics committees, hospital management or external governing bodies, with limited direct involvement of clinical leadership. Given that nursing and medical directors are responsible for key areas including strategic oversight, legal compliance, and professional supervision, these knowledge gaps may signal broader issues related to decentralised responsibility.^36^ If left unaddressed, this fragmentation could present risks to patient safety.^37^

In the absence of clear guidance for applying the Dying Decree Law, nursing and medical directors appear to have assumed the role of street-level bureaucrats: they interact directly with patients, have substantial discretion in their performance, and work in very complex and under-resourced environments. Simultaneously, they navigate a triangular field of responsibility towards staff, patients, and the institution itself, without the possibility of referring to a more appropriate authority. Providing clear procedural guidance, however, should be regarded as a core responsibility of health policy actors rather than the legislator, as it pertains to the practical governance of care provision rather than the creation of legal provisions.

Our findings underscore how, sandwiched between legal mandates and institutional practice, nursing and medical directors make judgement calls when navigating the Dying Decree Law. With no consistent governmental guidelines on implementation of the Dying Decree Law for healthcare institutions, nursing and medical directors become de facto self-reliant policymakers, shaping the law’s application in practice through varied interpretations of the legislation, resource constraints, and ethical/spiritual orientations.^38^ The substantial demand for additional legal (31.9%), ethical (30.2%), and psychological (17.5%) support underscores the burden of this accountability towards employees, patients, and institutions. This discretionary role places ethical and legal responsibility on individuals rather than institutions, further underscoring the need for consistent national-level implementation support.

As seen in the section “Encounters and experiences with assisted suicide,” the majority of nursing and medical directors with prior experience of assisted suicide cases in their institutions reported that relatives of PWTD were most often present during the process of establishing a dying decree.

This observation resonates with international findings which emphasise the vital role relatives play in supporting PWTD. While much country-specific assisted dying legislation does not mention relatives, in some countries it has become common practice to involve them.^39^ As relatives are often present, healthcare professionals have noted that the relatives are those needing greater support both before and after the patient’s death.^39–42^

The qualitative data reveal that in Austria, palliative care teams are often the first point of contact in response to assisted suicide requests. While many theorists argue that palliative care and assisted suicide are not aligned, many studies have shown that in practice, this is not the case.^7,43–46^ Evidence from Swiss palliative care settings, palliative care physicians are often deeply involved and engaged in cases of assisted suicide (Gamondi et al.^9^), suggesting a form of complementarity in certain contexts. At the same time, studies from Switzerland indicate that relatives of PWTD prefer to interact with Right To Die Societies rather than healthcare professionals. Across studies, a recurring theme is tension: palliative care teams navigate ethical and legal uncertainties while attempting to honour patient autonomy.^9,44,46^ This clearly highlights a need for support in clinical policies to help foster the intercorrelation of palliative care and assisted suicide.^41^ These findings also correlate with our participants’ reports: the information landscape is scarce and there is a lack of support, leaving medical and nursing directors in difficult situations as they try to navigate their teams around assisted suicide requests, following palliative care values while acting without clear juridical guidance and respecting patient autonomy. Overall, the literature and our data suggest that the relationship between palliative care and assisted suicide is context-dependent, shaped by institutional policies, team dynamics, and societal values and attitudes.

The findings on discrimination against and exclusion of PWTD with physical disabilities deserve particular attention. As shown in several participant comments, the Dying Decree Law lacks inclusivity and equity as the legislation currently excludes individuals with physical disabilities who are unable to take the final step unassisted – to hold the glass and drink, or to open the IV line. This raises serious ethical concerns regarding equity, non-discrimination and compliance with human rights frameworks. Similar issues have been observed internationally: in the United States, otherwise-eligible patients with neuromuscular disabilities are excluded from medical aid in dying if they cannot self-administer medications without assistance.^47^

By overlooking or excluding certain individuals in the legislative process, we risk perpetuating and exacerbating existing inequalities and inequities, thereby ensuring that such disparities persist not only throughout life but also at the end of life.^48^ The current Austrian legislation exempts certain people from accessing this possibility. We therefore emphasise the importance of ensuring inclusion and equity in matters of death and advocate for change in this regard. Future research should focus on optimising the current situation, particularly exploring how the legislation could be made more accessible.

### Strengths and limitations

The study addresses the widely debated and ethically complex issue of assisted suicide within healthcare institutions. Notably, it is the first study to record first-hand experiences and perspectives of nursing and medical directors following the establishment of the Dying Decree Law in Austria.

A particular strength of the study lies in its focus on nursing and medical directors in Austrian healthcare institutions. Their roles are central to institutional navigation but not explicitly mentioned in the legislation. By targeting this group, the study provides much needed insight into the practical and administrative challenges posed by the legislation. By integrating all types of healthcare institutions in all nine federal states of Austria that may be affected by the new law, this study is not only relevant for academic discourse on end-of-life care but also informs future policy and institutional guidelines.

While we have contacted various healthcare institutions from across Austria, participation was voluntary, so that nursing and medical directors with strong opinions on or personal experiences of assisted suicide may be overrepresented, displaying a potential study limitation. As the response rate was 19.1%, there are possible concerns regarding selection bias. Unfortunately, no population-level sociodemographic information is available for medical and nursing directors of Austrian healthcare institutions. Consequently, it is not possible to assess whether non-respondents differ systematically from respondents or to calculate differential response rates based on factors such as gender, age, tenure, etc. The Austrian healthcare system is fairly complex, and so it may not be possible to fully generalise the findings of this study to all institutional settings and healthcare professionals in the country. Furthermore, we did not assess the formal governance responsibilities of medical and nursing directors regarding development, which may differ across institutional settings.

Due to the anonymous study design and the absence of detailed information on non-respondents, systematic non-response patterns could not be assessed, and potential non-response bias cannot be excluded.

Another limitation of this study is that the questionnaire used was not formally validated, which may affect the reliability and interpretation of the findings. Moreover, another limitation of this study is that a formal power analysis was not conducted to calculate or justify the sample size, which may limit the ability to detect statistically significant effects.

Furthermore, this study was conducted in Q3 2024, representing a snapshot of the experiences and challenges faced by nursing and medical directors at a specific time, 2.5 years after the Dying Decree Law was established. Consequently, ongoing developments in policy discourse are not reflected in this study.

## Conclusion

The findings of this study highlight the importance of further research on assisted suicide in Austria and showcase the institutional response to adopting the legislation. They show that medical and nursing directors need further education, support, and guidance in responding to requests for and carrying out assisted suicides, particularly within healthcare institutions.

Such education, support, and guidance would enable medical and nursing directors to give the healthcare professionals in their institutions the tools they need to deal with assisted suicide requests, such as in-house guidelines, as well as helping them in their interactions with PWTD and their relatives.

Future research should focus on the effectiveness of the current guidelines; whether healthcare professionals consider the guidelines appropriate and applicable; how PWTD and their relatives experience these guidelines; and whether the guidelines adequately support end-of-life care.

Research into assisted suicide requests in healthcare institutions is essential to understand the barriers faced by the different parties involved, which in turn hinder compassionate care. Finally, research addressing the implementation of assisted suicide legislation and its practicability is highly relevant in the current political climate, as many countries are now voting on legalising assisted dying in some form. This research should ultimately inform the development of actionable institutional policies and national-level implementation guidelines.

## Supplemental Material

sj-docx-1-pcr-10.1177_26323524261436925 – Supplemental material for Institutional challenges in responding to Austria’s Dying Decree Law: An evaluation from the perspectives of nursing and medical directors Supplemental material, sj-docx-1-pcr-10.1177_26323524261436925 for Institutional challenges in responding to Austria’s Dying Decree Law: An evaluation from the perspectives of nursing and medical directors by Tamina-Laetitia Vielgrader, Jana Marica Hluch, Klara Doppler, Elisabeth Lucia Zeilinger, Ricarda Mewes, Sabine Parrag, Thomas Wochele-Thoma, Maria Kletecka-Pulker and Stefan Dinges in Palliative Care and Social Practice

sj-docx-2-pcr-10.1177_26323524261436925 – Supplemental material for Institutional challenges in responding to Austria’s Dying Decree Law: An evaluation from the perspectives of nursing and medical directors Supplemental material, sj-docx-2-pcr-10.1177_26323524261436925 for Institutional challenges in responding to Austria’s Dying Decree Law: An evaluation from the perspectives of nursing and medical directors by Tamina-Laetitia Vielgrader, Jana Marica Hluch, Klara Doppler, Elisabeth Lucia Zeilinger, Ricarda Mewes, Sabine Parrag, Thomas Wochele-Thoma, Maria Kletecka-Pulker and Stefan Dinges in Palliative Care and Social Practice

sj-docx-3-pcr-10.1177_26323524261436925 – Supplemental material for Institutional challenges in responding to Austria’s Dying Decree Law: An evaluation from the perspectives of nursing and medical directors Supplemental material, sj-docx-3-pcr-10.1177_26323524261436925 for Institutional challenges in responding to Austria’s Dying Decree Law: An evaluation from the perspectives of nursing and medical directors by Tamina-Laetitia Vielgrader, Jana Marica Hluch, Klara Doppler, Elisabeth Lucia Zeilinger, Ricarda Mewes, Sabine Parrag, Thomas Wochele-Thoma, Maria Kletecka-Pulker and Stefan Dinges in Palliative Care and Social Practice

sj-docx-4-pcr-10.1177_26323524261436925 – Supplemental material for Institutional challenges in responding to Austria’s Dying Decree Law: An evaluation from the perspectives of nursing and medical directors Supplemental material, sj-docx-4-pcr-10.1177_26323524261436925 for Institutional challenges in responding to Austria’s Dying Decree Law: An evaluation from the perspectives of nursing and medical directors by Tamina-Laetitia Vielgrader, Jana Marica Hluch, Klara Doppler, Elisabeth Lucia Zeilinger, Ricarda Mewes, Sabine Parrag, Thomas Wochele-Thoma, Maria Kletecka-Pulker and Stefan Dinges in Palliative Care and Social Practice

sj-docx-5-pcr-10.1177_26323524261436925 – Supplemental material for Institutional challenges in responding to Austria’s Dying Decree Law: An evaluation from the perspectives of nursing and medical directors Supplemental material, sj-docx-5-pcr-10.1177_26323524261436925 for Institutional challenges in responding to Austria’s Dying Decree Law: An evaluation from the perspectives of nursing and medical directors by Tamina-Laetitia Vielgrader, Jana Marica Hluch, Klara Doppler, Elisabeth Lucia Zeilinger, Ricarda Mewes, Sabine Parrag, Thomas Wochele-Thoma, Maria Kletecka-Pulker and Stefan Dinges in Palliative Care and Social Practice

sj-docx-6-pcr-10.1177_26323524261436925 – Supplemental material for Institutional challenges in responding to Austria’s Dying Decree Law: An evaluation from the perspectives of nursing and medical directors Supplemental material, sj-docx-6-pcr-10.1177_26323524261436925 for Institutional challenges in responding to Austria’s Dying Decree Law: An evaluation from the perspectives of nursing and medical directors by Tamina-Laetitia Vielgrader, Jana Marica Hluch, Klara Doppler, Elisabeth Lucia Zeilinger, Ricarda Mewes, Sabine Parrag, Thomas Wochele-Thoma, Maria Kletecka-Pulker and Stefan Dinges in Palliative Care and Social Practice

## References

[1] Neves MFA . Politics and practices of assisted dying. In: Clark D Samuels A (eds) Research handbook on end of life care | society. Edward Elgar Publishing, 2025, pp. 304–18, https://www.elgaronline.com/edcollchap-oa/book/9781035317349/chapter19.xml (accessed 7 January 2026).

[2] Cholbi M Varelius J (eds). New directions in the ethics of assisted suicide and euthanasia [Internet]. The International Library of Bioethics, Volume 103. Springer International Publishing, https://link.springer.com/10.1007/978-3-031-25315-7 (2023, accessed 7 January 2026)

[3] WFRTDS. Austria – The World Federation of Right to Die Societies [Internet], https://wfrtds.org/worldmap/austria/ (n.d., 25 June 2025).

[4] Espericueta L. Analysis of the legal situation regarding euthanasia in Ecuador, Colombia, and Peru: towards a Latin American model of medical assistance in dying? Dev World Bioeth 2025; 25(2): 98–104. 38995203 10.1111/dewb.12457

[5] White B (ed.). Research handbook on voluntary assisted dying: law, regulation and practice [Internet]. Edward Elgar Publishing, https://e-elgar.com/shop/usd/research-handbook-on-voluntary-assisted-dying-law-regulation-and-practice-9781802204346.html?srsltid=AfmBOorBdXo1C5JgxVB3Vz7NXs5fjHt7IBUjoMp-eWSaKXqhxomYyiG9 (2025, accessed January 9 2026).

[6] Angelika Feichtner. Assistierter Suizid in Österreich – erste Erkenntnisse aus ASCIRS [Internet], https://magazin.pflegenetz.at/artikel/assistierter-suizid-in-oesterreich-erste-erkenntnisse-aus-ascirs/ (2025, accessed 7 October 2025).

[7] Bergenholtz H Timm HU Missel M. Talking about end of life in general palliative care – what’s going on? A qualitative study on end-of-life conversations in an acute care hospital in Denmark. BMC Palliat Care 2019; 18(1): 62. 31345196 10.1186/s12904-019-0448-z PMC6657144

[8] Bergenholtz H Missel M Timm H. Talking about death and dying in a hospital setting: a qualitative study of the wishes for end-of-life conversations from the perspective of patients and spouses. BMC Palliat Care 2020; 19(1): 168. 33138799 10.1186/s12904-020-00675-1 PMC7607873

[9] Gamondi C Borasio GD Oliver P , et al. Responses to assisted suicide requests: an interview study with Swiss palliative care physicians. BMJ Support Palliat Care 2019; 9(1): e7. 10.1136/bmjspcare-2016-001291 28801317

[10] Sandham M Carey M Hedgecock E , et al. Nurses’ experiences of supporting patients requesting voluntary assisted dying: a qualitative meta-synthesis. J Adv Nurs 2022; 78(10): 3101–3115. 35748092 10.1111/jan.15324 PMC9546017

[11] Wilson M Ostroff C Wilson ME , et al. Profiles of intended responses to requests for assisted dying: a cross-sectional study. Int J Nurs Stud 2021; 124: 104069. 34592533 10.1016/j.ijnurstu.2021.104069

[12] Lenhart A Weixler D Schaden E , et al. Assistierter Suizid ist noch keine “area of interest” für Ärzt:innen. Anästhesie Nachr 2024; 6(1): 1–14.

[13] Kitta A Ecker F Zeilinger EL , et al. Statements of Austrian hospices and palliative care units after the implementation of the law on assisted suicide: a qualitative study of web-based publications. Wien Klin Wochenschr 2024; 136(13–14): 382–389. 36894787 10.1007/s00508-023-02157-9 PMC11239715

[14] Fischer L Le NS Kirchner S , et al. Evaluating physicians’ knowledge of assisted suicide and palliative sedation therapy in Austria: an exploratory survey study. Wien Klin Wochenschr 2025; 137: 357–367. 10.1007/s00508-025-02509-7 PMC12177003 40035840

[15] Masel EK. Perspective: legal, ethical, and medical perspectives of the landscape of assisted suicide in Austria. Wien Klin Wochenschr 2024; 136(13–14): 380–381. 38530423 10.1007/s00508-024-02344-2

[16] Stolz E Mayerl H Gasser-Steiner P , et al. Attitudes towards assisted suicide and euthanasia among care-dependent older adults (50+) in Austria: the role of socio-demographics, religiosity, physical illness, psychological distress, and social isolation. BMC Med Ethics 2017; 18(1): 71. 29212490 10.1186/s12910-017-0233-6 PMC5719645

[17] Gastmans C Lemiengre J de Casterlé BD. Development and communication of written ethics policies on euthanasia in Catholic hospitals and nursing homes in Belgium (Flanders). Patient Educ Couns 2006; 63(1): 188–195. 16406462 10.1016/j.pec.2005.10.004

[18] Gastmans C Lemiengre J van der Wal G , et al. Prevalence and content of written ethics policies on euthanasia in Catholic healthcare institutions in Belgium (Flanders). Health Policy 2006; 76(2): 169–178. 16221504 10.1016/j.healthpol.2005.09.003

[19] Lemiengre J Dierckx de Casterlé B Schotsmans P , et al. Written institutional ethics policies on euthanasia: an empirical-based organizational-ethical framework. Med Health Care Philos 2014; 17(2): 215–228. 24420744 10.1007/s11019-013-9524-y

[20] Zeilinger EL Vielgrader TL Petersen A , et al. Nurses’ perspectives on assisted suicide: challenges and support needs. Soc Sci Med 2025; 366: 117663. 39740633 10.1016/j.socscimed.2024.117663

[21] Zeilinger EL Petersen A Brunevskaya N , et al. Experiences and attitudes of nurses with the legislation on assisted suicide in Austria. Palliat Support Care 2024; 22(6): 2022–2028. 10.1017/S147895152400107X 39390951

[22] Unseld M Meyer AL Vielgrader TL , et al. Assisted suicide in Austria: nurses’ understanding of patients’ requests and the role of patient symptoms. Int J Environ Res Public Health 2025; 22(2): 218. 40003444 10.3390/ijerph22020218 PMC11855785

[23] Bundesministerium für Arbeit Soziales Gesundheit Pflege undKonsumentenschutz (BMASGPK). Sterbeverfügung-Leitfadenfür die Praxis, https://www.google.com/url?sa=tsource=webrct=jopi=89978449url=https://www.sozialministerium.gv.at/dam/jcr:41404f4e-adc6-40da-a69a-bc30168b85c4/Sterbeverf%25C3%25BCgung_Leitfaden_f%25C3%25BCr_die_Praxis_-_Version_04.2025.pdf&ved=2ahUKEwj9pvjg0umOAxXBVfEDHY3iKfIQFnoECBUQAQ&usg=AOvVaw3pZp1JpEt6VR8v_o8C1NZZ (2025, accessed 26 February 2025).

[24] Hamer MK Baugh CM Bolcic-Jankovic D , et al. Conscience-based barriers to medical aid in dying: a survey of colorado physicians. J Gen Intern Med 2024; 39(16): 3138–3145. 38710866 10.1007/s11606-024-08782-y PMC11618266

[25] Kraak-Steenken FWM Renckens SC Pasman HRW , et al. Euthanasia and physician-assisted suicide in people with an accumulation of health problems related to old age: a cross-sectional questionnaire study among physicians in the Netherlands. Int J Public Health 2024; 69: 1606962. 38698912 10.3389/ijph.2024.1606962 PMC11064696

[26] Pottash M Saikaly K Stevenson M , et al. A survey of clinicians who provide aid in dying. Am J Hosp Palliat Care 2024; 41(9): 1045–1050. 37776055 10.1177/10499091231205841

[27] Smets T Cohen J Bilsen J , et al. Attitudes and experiences of Belgian physicians regarding euthanasia practice and the euthanasia law. J Pain Symptom Manage 2011; 41(3): 580–593. 21145197 10.1016/j.jpainsymman.2010.05.015

[28] Bundesministerium für Arbeit Soziales Gesundheit Pflege undKonsumentenschutz. Startpage | Federal Ministry – Labour Social Affairs Health Care Consumer Protection, https://www.sozialministerium.gv.at/en.html (accessed 22 May 2025).

[29] GÖG. Front page | Gesundheit Österreich GmbH [Internet], https://goeg.at/en (accessed 22 May 2025).

[30] Leiner D. SoSci Survey [Internet], https://www.soscisurvey.de (accessed 3 June 2025).

[31] JASP. https://jasp-stats.org (2024, accessed 25 June 2025).

[32] Braun V Clarke V. Using thematic analysis in psychology. Qual Res Psychol 2006; 3(2): 77–101.

[33] MAXQDA. https://www.maxqda.com/de/ (2021, accessed 25 June 2025).

[34] Ewick P Silbey SS. The common place of law: stories from everyday life. University of Chicago Press, 1998, p. 318 (Language and legal discourse).

[35] Dialogforum Sterbehilfe. https://www.bmvrdj.gv.at/service/publikationen/Dialogforum-Sterbehilfe.html (2021, accessed 18 July 2025).

[36] Blume LHK van Weert NJHW Busari JO , et al. Guideline adherence: how do boards of directors deal with it? A survey in Dutch hospitals. J Hosp Adm 2016; 5(5): 21–29.

[37] Joshi MS Hines SC. Getting the board on board: engaging hospital boards in quality and patient safety. Jt Comm J Qual Patient Saf 2006; 32(4): 179–187. 16649648 10.1016/s1553-7250(06)32023-5

[38] Lipsky M. Street level bureaucracy: dilemmas of the individual in public services [Internet]. Russell Sage Foundation, https://www.jstor.org/stable/10.7758/9781610447713 (1980, accessed 25 June 2025).

[39] Gamondi C Fusi-Schmidhauser T Oriani A , et al. Family members’ experiences of assisted dying: A systematic literature review with thematic synthesis. Palliat Med 2019; 33(8): 1091–1105. 31244384 10.1177/0269216319857630

[40] Boven C Dillen L Dierickx S , et al. Relatives’ experiences of being involved in assisted dying: a qualitative study. Qual Health Res 2023; 33(13): 1154–1164. 37791685 10.1177/10497323231196827 PMC10626978

[41] Gamondi C Pott M Preston N , et al. Swiss families’ experiences of interactions with providers during assisted suicide: a secondary data analysis of an interview study. J Palliat Med 2020; 23(4): 506–512. 31697177 10.1089/jpm.2019.0286

[42] Renckens SC Onwuteaka-Philipsen BD van der Heide A , et al. Physicians’ views on the role of relatives in euthanasia and physician-assisted suicide decision-making: a mixed-methods study among physicians in the Netherlands. BMC Med Ethics 2024; 25(1): 43. 38580964 10.1186/s12910-024-01031-1 PMC10996154

[43] Chochinov HM. Getting MAD (Medical Aid in Dying) in Canada. Palliat Support Care 2014; 12(6): 423–424. 25997936 10.1017/S1478951514001400

[44] Dierickx S Deliens L Cohen J , et al. Involvement of palliative care in euthanasia practice in a context of legalized euthanasia: a population-based mortality follow-back study. Palliat Med 2018; 32(1): 114–122. 28849727 10.1177/0269216317727158 PMC5758933

[45] Downar J MacDonald S Buchman S. Medical assistance in dying and palliative care: shared trajectories. J Palliat Med 2023; 26(7): 896–899. 37428971 10.1089/jpm.2023.0209

[46] Michael N Jones D Kernick L , et al. Does voluntary assisted dying impact quality palliative care? A retrospective mixed-method study. BMJ Support Palliat Care. Epub ahead of print 14 September 2025. DOI: 10.1136/spcare-2024-004946. 38871403

[47] Shavelson L Pope TM Battin MP , et al. Neurologic diseases and medical aid in dying: aid-in-dying laws create an underclass of patients based on disability. Am J Bioeth 2023; 23(9): 5–15. 10.1080/15265161.2022.2105422 PMC9931932 35972304

[48] Riddle CA. Assisted dying & disability. Bioethics 2017; 31(6): 484–489. 28419505 10.1111/bioe.12353

