# Supplementary material for: Institutional challenges in responding to Austria’s Dying Decree Law: An evaluation from the perspectives of nursing and medical directors
Source: Palliat Care Soc Pract. 2026 Apr 26;20:26323524261436925. doi: 10.1177/26323524261436925 (PMC13129360; doi:10.1177/26323524261436925)
Supplement: sj-docx-1-pcr-10.1177_26323524261436925 – Supplemental material for Institutional challenges in responding to Austria’s Dying Decree Law: An evaluation from the perspectives of nursing and medical directors [file sj-docx-1-pcr-10.1177_26323524261436925.docx]

**Online Questionnaire**

Target Group: Nursing and Medical Directors

**Dear participants of this survey,**

The Institute for Ethics and Law in Medicine at the University of Vienna is conducting a survey for a multi-perspective evaluation of the **Dying Decree Law** (*Sterbeverfügungsgesetz*). The aim of this study is to identify current issues arising in the implementation of the new **Dying Decree Law** and, based on ethical, legal, and social science analyses, to develop appropriate solution perspectives.

Answering all questions will take approximately 10–15 minutes.
In order to describe the sample of respondents more precisely, we will also collect sociodemographic data (age, gender, employment status) in anonymized form (it will not be possible to draw conclusions about your person). These questions are optional; however, complete responses are important, as this information significantly contributes to a comprehensive understanding of how dying decrees are handled in Austria.

Only specifically marked and voluntarily provided information – such as the name and location of your workplace – will be analyzed separately and may be skipped. All other data will be anonymized and used exclusively for research purposes in compliance with the GDPR.

Participation in this study is entirely voluntary. You may withdraw from participation at any time without giving any reason. The survey does not contain any data that could identify you as an individual, thus your anonymity is safeguarded. All data collected in this study will be treated with strict confidentiality. Data will be analyzed only in anonymized, group-related form for scientific purposes; no individual-related evaluation will take place. The research is conducted without any commercial interest. All your data will be handled with the utmost confidentiality.

If you have any questions about the content, purpose, or research ethics of this study, please contact tamina-laetitia.vielgrader@univie.ac.at or ierm@univie.ac.at.

We sincerely thank you for participating in this survey and supporting us with your responses!

**By clicking “Next,” you confirm that you have understood the information about participating in the survey and that you agree to take part.**

**1) In which federal state is your institution/practice located?**
[Multiple Choice]

- Vienna
- Lower Austria
- Burgenland
- Upper Austria
- Salzburg
- Carinthia
- Styria
- Tyrol
- Vorarlberg

**2) In what type of institution do you work**
[Multiple Choice]

- Hospital operated by a public provider
- Hospital operated by a private provider
- Care facility operated by a public provider
- Care facility operated by a private provider
- Other: __________

**3) In what type of institution are you employed?**
[Single choice]

- Faith-based
- Non-faith-based
- None of the above

**4) How long (in years, rounded up) have you been working in this institution?**
[Open text field]
________ years

**5) Approximately how many patients/clients does your institution care for annually (rough estimate)?**
[Open question]

**6) How old are you?**

[Open text field]

_________Years

**7)** **What is your gender identity?**
[Single choice]

- Female
- Male
- Diverse
- Open
- Prefer not to say

**8)** **Are you familiar with the new regulations of the Dying Decree Law (abbreviated: StVfG)?**
[Single choice]

- Yes, I am familiar with the regulations and can apply them
- Yes, I am familiar with the regulations, but I am unsure about applying them
- No, I am not familiar with the regulations

**9) Have patients/clients ever expressed a wish to establish a Dying Decree during their stay at your institution?**
[Single choice]

- Yes Jump to: 9.2
- No Jump to: 10
- I don’t know Jump to: 10

**9.2 Since the entry into force of the Dying Decree Law (01/01/2022), how often have patients/clients expressed a wish to establish a Dying Decree?**
[Single choice]

- 1–3
- 4–6
- 7–10
- More than 10

**9.3 In which setting was the wish to establish a Dying Decree expressed?**
[Multiple choice]

- Hospital operated by a public provider
- Hospital operated by a private provider
- Care facility operated by a public provider
- Care facility operated by a private provider
- In the private setting of the person wishing to die
- Other: __________

**9.4** **Has any type of support for patients/clients who expressed a wish for assisted suicide been provided by your institution or by interest groups (e.g., the Medical Association)?**
[Single choice]

- Yes, namely: __________
- No

**10) “Since the entry into force of the Dying Decree Law (01/01/2022), have you encountered any patients/clients in your institution with a legally valid Dying Decree?”**
[Single choice]

- Yes    Jump to: 10.2
- No    Jump to: 11
- I don’t know   Jump to: 11

**10.2** **Since the entry into force of the Dying Decree Law (01/01/2022), how many patients/clients with a legally valid Dying Decree have you encountered?**
[Single choice]

- 1–3
- 4–6
- 7–10
- More than 10

**11) Has a patient/client in your institution ever expressed a wish to carry out assisted suicide with a legally valid Dying Decree?**
[Single choice]

- Yes    Jump to: 11.2
- No    Jump to: 12
- I don’t know    Jump to: 12

**11.2) Since the entry into force of the Dying Decree Law (01/01/2022), how often has a patient/client expressed a wish to actively carry out assisted suicide with a Dying Decree?**
[Single choice]

- 1–3
- 4–6
- 7–10
- More than 10

**11.3) How was this wish addressed?**
[Multiple choice]

- Assisted suicide was carried out with the patient/client to the best possible extent
- Patient/client was sent home to carry out assisted suicide
- Wish for assisted suicide was supported (not yet carried out) by facilitating contacts
- Wish for assisted suicide was declined
- Offer of further clarifying discussions
- Involvement of psychological/psychiatric consultations
- I don’t know

**11.4)** **Were any other persons – apart from the helper named in the Dying Decree and the medical professional – involved in carrying out the Dying Decree?**
[Multiple choice]

- Representative of health professions (from the same healthcare institution)
- Representative of health professions (external to the healthcare institution)
- Relatives of the person wishing to die
- Other persons (specify) ____

**11.5)** **Do you consider the handling of assisted suicides within the framework of Dying Decrees that you have experienced so far to be appropriate?**
[Likert scale]

- Strongly agree
- Somewhat agree
- Neutral
- Somewhat disagree
- Strongly disagree

**12)** **Does your institution have a guideline for handling existing Dying Decrees and/or requests for support in establishing a Dying Decree?**
[Single choice]

- Yes    Jump to: 13
- No    Jump to: 12.2
- I don’t know   Jump to: 14

**12.2“If applicable, is the development of such a guideline planned?”**[Open question]    Jump to: 14

**13) How helpful do you find this guideline?**
[7-point scale]
Very helpful - - - - - Not helpful at all

**14) In your view, is there a specific need for changes regarding the implementation of the Dying Decree Law in daily practice?**
[Single choice]

- Yes, namely: __________
- No

**15) Would you like to receive more information and guidance on the Dying Decree Law?**
[Multiple choice]

- Yes, more legal information and details would be helpful.
- Yes, more ethical guidelines would be helpful.
- Yes, more psychological support would be helpful.
- No, I already have all the necessary information.

**16) What is the name of the institution where you work, and what is the name of the organization that operates it?”**
[Open text field]

Name of institution: __________
Name of organization/operator: __________

**17) As part of the study evaluating the Dying Decree Law, qualitative interviews with experts are being conducted. If you are generally interested in participating in such an interview, please feel free to leave an email address for contact!**[Open question]

Email address: __________

**You have now reached the end of the questionnaire.
If you have any questions about the content, purpose, or research ethics of this study, please contact** [**tamina-laetitia.vielgrader@univie.ac.at**](mailto:tamina-laetitia.vielgrader@univie.ac.at) **or** [**ierm@univie.ac.at**](mailto:ierm@univie.ac.at)**.**

**Thank you for your participation. You may now close your web browser.**
