# Supplementary material for: Institutional challenges in responding to Austria’s Dying Decree Law: An evaluation from the perspectives of nursing and medical directors
Source: Palliat Care Soc Pract. 2026 Apr 26;20:26323524261436925. doi: 10.1177/26323524261436925 (PMC13129360; doi:10.1177/26323524261436925)
Supplement: sj-docx-2-pcr-10.1177_26323524261436925 – Supplemental material for Institutional challenges in responding to Austria’s Dying Decree Law: An evaluation from the perspectives of nursing and medical directors [file sj-docx-2-pcr-10.1177_26323524261436925.docx]

*Additional File 2. Distribution of knowledge and confidence levels regarding the Dying Decree Law by gender, religious affiliation, and institution type ^^[[1]](#footnote-1)^^,^^[[2]](#footnote-2)^^*

| **Knowledge and understanding of the Dying Decree Law** | **Gender** | | | **Religious affiliation** | | | **Type of institution** | | | | |  |
| --- | --- | --- | --- | --- | --- | --- | --- | --- | --- | --- | --- | --- |
|  | w | m | total | faith-based | non-faith-based | total | public hospital | private hospital | public nursing home | private nursing home | total | |
| Knowledge and confidence in Applying | *n*=67 (59.3%) | *n*=46 (40.7%) | *n*=113 | *n*=35 (34.3%) | *n*=67 (65.7%) | *n*=102 | *n*=30 (27.8%) | *n*=19 (17.6%) | *n*=37  (34.2%) | *n*=22  (20.4%) | *n*=108 | |
| Knowledge without confidence in applying | *n*=70 (77.8%) | *n*=20 (22.2%) | *n*=90 | *n*=19 (26.0%) | *n*=54 (74.0%) | *n*=73 | *n*=20 (23.2%) | *n*=9 (10.5%) | *n*=43  (50.0%) | *n*=14  (16.3%) | *n*=86 | |
| No knowledge | *n*=20 (64.5%) | *n*=11 (35.5%) | *n*=31 | *n*=2 (7.4%) | *n*=25 (92.6%) | *n*=27 | *n*=14 (46.6%) | *n*=2  (6.7%) | *n*=8  (26.7%) | *n*=6  (20.0%) | *n*=30 | |
| **X2-tests for significance** | **Test Statistic** | ***p*-value** | **Effect Size** | **Test Statistic** | ***p*-value** | **Effect Size** | **Test Statistic** | ***p*-value** | **Effect Size** |  | |  |
|  | *χ²*(2) = 7.862 | .020* | *V* = 0.183 | *χ²*(2) = 7.877 | .019* | *V* = 0.197 | *χ²*(6) = 12.178 | .058 | *V* = 0.165 |  | |  |

1. Percentages refer to the subgroup of those who reported knowledge of the Dying Decree Law (*n*=207), not to the total sample (*N*=239). [↑](#footnote-ref-1)
2. Totals may differ due to missing or incomplete data. [↑](#footnote-ref-2)
