# Supplementary material for: Institutional challenges in responding to Austria’s Dying Decree Law: An evaluation from the perspectives of nursing and medical directors
Source: Palliat Care Soc Pract. 2026 Apr 26;20:26323524261436925. doi: 10.1177/26323524261436925 (PMC13129360; doi:10.1177/26323524261436925)
Supplement: sj-docx-3-pcr-10.1177_26323524261436925 – Supplemental material for Institutional challenges in responding to Austria’s Dying Decree Law: An evaluation from the perspectives of nursing and medical directors [file sj-docx-3-pcr-10.1177_26323524261436925.docx]

Additional File 3. Group differences regarding gender, religious affiliation and type of institution

| **Knowledge of the Death Decree Act** | | | | |
| --- | --- | --- | --- | --- |
| **Variable** | **Test Statistic** | **df** | ***p*-value** | **Effect Size** |
| gender | *χ²*(2) = 7.862 | 2 | .020 | *V* =0.183 |
| religious affiliation | *χ²*(2) = 7.877 | 2 | .019 | *V* = 0.197 |
| type of institution | *χ²*(6) = 12.178 | 6 | .058 | *V* = 0.165 |
|  |  |  |  |  |
| **Guidelines (issuing)** | | | | |
| **Variable** | **Test Statistic** | **df** | ***p*-value** | **Effect Size** |
| gender | *χ²*(1) = 0.812 | 1 | .368 | *V* *=* 0.065 |
| religious affiliation | *χ²*(1) = 3.507 | 1 | .061 | *V* = 0.146 |
| type of institution | *χ²*(3) = 7.363 | 3 | .061 | *V* = 0.202 |
|  |  |  |  |  |
| **Guideline Satisfaction** | | | | |
| **Variable** | **Test Statistic** | **df** | ***p*-value** | **Effect Size** |
| gender | *U* = 1772.000 |  | .004 | *r* = 0.15 |
| religious affiliation | *U* = 705.500 |  | .003 | *r* = -0.15 |
| type of institution | *F* = 4.178 | 3, 45.785 | .011 | *η²* = 0.126 |
