# Supplementary material for: Institutional challenges in responding to Austria’s Dying Decree Law: An evaluation from the perspectives of nursing and medical directors
Source: Palliat Care Soc Pract. 2026 Apr 26;20:26323524261436925. doi: 10.1177/26323524261436925 (PMC13129360; doi:10.1177/26323524261436925)
Supplement: sj-docx-4-pcr-10.1177_26323524261436925 – Supplemental material for Institutional challenges in responding to Austria’s Dying Decree Law: An evaluation from the perspectives of nursing and medical directors [file sj-docx-4-pcr-10.1177_26323524261436925.docx]

Additional File 4. Types of support

| **Themes** | **Quotes** |
| --- | --- |
| 1. Guidance/Support | -P67, „Social support for the affected person and their relatives; conversations. “ |
|  | -P59, “In-house social worker” |
|  | -P230, „Conversation with an ethics consultant” |
|  | -P108 „spiritual support and counseling “ |
| 1. Counseling by (psy) professions | -P219, “Conversation with psychologists and psychiatrists” |
|  | -P176, “Psychologists, general practitioners“ |
| 1. External advisory/Information services | -P148, “Website of the Medical Chamber; conversation about the possibility of palliative care (no support for assisted suicide)” |
|  | -P154, “Conversation, referral to designated counseling centers” |
|  | -P71, “Information centers mentioned“ |
| 1. Inclusion of/Referral to external health professional | -P192, “General practitioner, ongoing communication with the relatives” |
|  | -P101, “General practitioner and hospice team” |
| 1. Legal experts | -P92, “Referral to the patients' ombudsperson” |
|  | -P226, “Palliative care, counseling by the Upper Austrian Patient Advocacy Service” |
|  | -P231, “Clarification of the legal situation” |
| 1. Palliative Care | -P172, “Palliative counseling and therapy services, multiprofessional support“ |
|  | -P130, “Taking the wish seriously and exploring its background, palliative care and counseling“ |
