# Supplementary material for: Institutional challenges in responding to Austria’s Dying Decree Law: An evaluation from the perspectives of nursing and medical directors
Source: Palliat Care Soc Pract. 2026 Apr 26;20:26323524261436925. doi: 10.1177/26323524261436925 (PMC13129360; doi:10.1177/26323524261436925)
Supplement: sj-docx-5-pcr-10.1177_26323524261436925 – Supplemental material for Institutional challenges in responding to Austria’s Dying Decree Law: An evaluation from the perspectives of nursing and medical directors [file sj-docx-5-pcr-10.1177_26323524261436925.docx]

Additional File 5. Needed changes to the legislation

| **Themes** | **Quotes** |
| --- | --- |
| 1. Practicability of the law, lack of clarity | -P3, „too complicated “ |
|  | -P137, “General simplification (the current regulation is difficult to understand); lockable boxes for the medication (currently not available)” |
|  | -P127, „It must be feasible in practice.” |
|  | -P60 „Compatibility between theory and practice “ |
| 1. Responsibility | -P202, “Processes are complex, with little information on who can provide support.” |
|  | -P79, “Establish a clear point of contact that provides legal clarification and information.“ |
| 1. Bureaucratic administration | -P46, “less bureaucracy” |
|  | -P36, “It should not intended to be applicable to nursing homes due to the severe overload resulting from inadequate staffing levels” |
| 1. Economic impact | -P35, “Easier access and cost reduction” |
|  | -P52, “Costs and elaborate expert reports; possibility for individuals with mental illnesses.” |
|  |  |
| 1. Discrimination | -P183, “The intake must be carried out personally, leading to discrimination against physically disabled individuals “ |
|  | -P45, “The intake of the medication is problematic: the patient may drink it incompletely or have a swallowing disorder.“ |
